# Comparison of postoperative outcomes between patients with positive and negative straight leg raising tests who underwent full-endoscopic transforaminal lumbar discectomy

**DOI:** 10.1038/s41598-020-73357-w

**Published:** 2020-10-05

**Authors:** Fei-long Wei, Haoran Gao, Xiaodong Yan, Yifang Yuan, Shu Qian, Quanyou Gao, Shikong Guo, Weigao Xue, Jixian Qian, Chengpei Zhou

**Affiliations:** Department of Orthopedics, Tangdu Hospital, The Fourth Military Medical University, 569 Xinsi Road, Xi’an, 710038 Shaanxi People’s Republic of China

**Keywords:** Diseases, Medical research

## Abstract

Full-endoscopic transforaminal lumbar discectomy (FETD) is increasingly used in patients with lumbar disc herniation (LDH). There is little knowledge on the related factors, including the straight leg raising test (SLR), that influence the operation. Consecutive patients with LDH who came to our hospital from August 2015 to September 2016 and underwent FETD surgery were included. Four kinds of scores, including the VAS (lumbar/leg), ODI and JOA values, were measured and reassessed after FETD to assess the surgical outcomes. There was a statistically significant difference between the scores before surgery and at each postoperative follow-up. In addition, the increase in the JOA score postoperatively was statistically significant compared with that before surgery. There were statistically significant differences among the three subpopulations [patients considered SLR positive (0°–30°), SLR positive (31°–60°) and SLR negative (61°–)] in the changes in the VAS (leg), ODI and JOA values. However, there were no statistically significant differences among the three subpopulations [patients considered SLR positive (0°–30°), SLR positive (31°–60°) and SLR negative (61°–)] in the changes in VAS score (lumbar). FETD showed great effectiveness in treating patients with lumbar disc herniation. Patients who were SLR negative may receive greater benefit from FETD.

## Introduction

Lumbar disc herniation (LDH) is one of the most common disorders, with a prevalence of approximately 77.8%^1,2^. LDH is mainly due to the compression of nerves by the nucleus pulposus, annulus fibrosus and cartilage plate, but especially the nucleus pulposus. After degenerative changes of the lumbar vertebra occur in different degrees, the intervertebral disc annulus fibrosus is broken under the action of external factors, and the nucleus pulposus enters the posterior vertebral canal, which then leads to stimulation or compression of the nerve roots of the adjacent spinal cord. Then, a series of clinical symptoms, such as lumbar pain, numbness and pain in one lower limb or both lower limbs, occur.

Traditional open lumbar microdiscectomy (OLM) is considered the gold standard of LDH treatment for its good efficacy in long-term follow-up^3,4^. In recent years, minimally invasive techniques have developed rapidly, and minimally invasive discectomy (MID) has been gradually applied to treat lumbar intervertebral disc herniation; this method is more minimally invasive and conducive to postoperative rehabilitation than open surgery^5^. Novel MID procedures have many potential advantages over standard microdiscectomy or open discectomy (MD/OD), including less blood loss, less postoperative pain, shorter hospitalization periods and an earlier return to work^6^.

The straight leg raising (SLR) test is a common and valuable examination that can reflect the severity of lumbar disc herniation and the degree of nerve root compression to some extent^7^. Jonsson’s research showed that the SLR test has a strong correlation with various parameters reflecting the degree of pain. A positive postoperative SLR test was associated with an inferior outcome^8^. However, there are few studies on the related factors, including the SLR test, that influence the operation, and there are few reports on which type of patients will benefit more from this kind of operation. Therefore, we designed this prospective study to explore the relevant factors influencing the postoperative effect of full-endoscopic transforaminal lumbar discectomy (FETD) surgery^9^ to provide a reference for clinical diagnosis and treatment. In this study, we collected preoperative and postoperative data, including the visual analogue scale (VAS) score, Oswestry Disability Index and Japanese Orthopaedic Association (JOA) score, of the patients and analysed the relationship between these scores and SLR to determine the influence of SLR on the postoperative clinical results. This study provides a reference for the clinical evaluation of surgical indications and contraindications.

## Methods

### Patients and follow-up

After obtaining consent from the hospital ethics committee, all patients provided written informed consent to participate in the prospective cohort study. All experiments were performed in accordance with relevant guidelines and regulations. According to the strict inclusion and exclusion criteria, we selected 118 consecutive patients with LDH who came to our hospital from August 2015 to September 2016 and underwent FETD surgery. Only 96 of these patients had complete data and were followed up for three years. Their surgical outcomes were assessed using the VAS (lumbar/leg), ODI and JOA. Patients completed these assessments one day before surgery. Four kinds of scores, including the VAS score (lumbar/leg), ODI and JOA score, were measured and reassessed 1 day, 3 months, 6 months, 12 months and 36 months after FETD. Our initial hypothesis was that the differences in the 4 scores between SLR-positive and SLR-negative patients would be statistically significant. The operation was performed by two doctors with more than 10 years of spinal neurosurgery experience and had received professional training in spinal endoscopy.

### Inclusion criteria

(1) Pain in the lower back with radiating pain and/or painful numbness in the unilateral or bilateral lower limbs; (2) physical signs and symptoms consistent with those on physical examination and are located in the same responsible segment; (3) X-ray computed tomography (CT), magnetic resonance imaging (MRI) and other imaging examinations confirmed that the responsible segment was consistent with the symptoms and signs, showing compression of the nerve root or dural sac with the appearance of a herniation disc in a single segment; (4) regular conservative treatment (medication, rehabilitation) for 8 weeks was not effective or led to worsened symptoms; (5) postoperative follow-up of 36 months and complete follow-up data ; and (6) age older than 18.

### Exclusion criteria

(1) Lower lumbar and lower limb pain with no apparent cause; (2) multilevel cervical disc herniation; (3) responsible segment is associated with prominent posterior or lateral protrusion deformities, extensive calcification of the intervertebral disc, or loss of intervertebral height; (4) responsible section had undergone interventional treatment, posterior excision and other surgical treatment; (5) spinal tuberculosis, infection, tumour, etc.; (6) diagnosis of lumbar spinal stenosis, lumbar instability or spondylolisthesis; and (7) cardiopulmonary diseases, coagulopathy, mental diseases and other surgical contraindications.

### Surgical techniques

**Figure 1:**
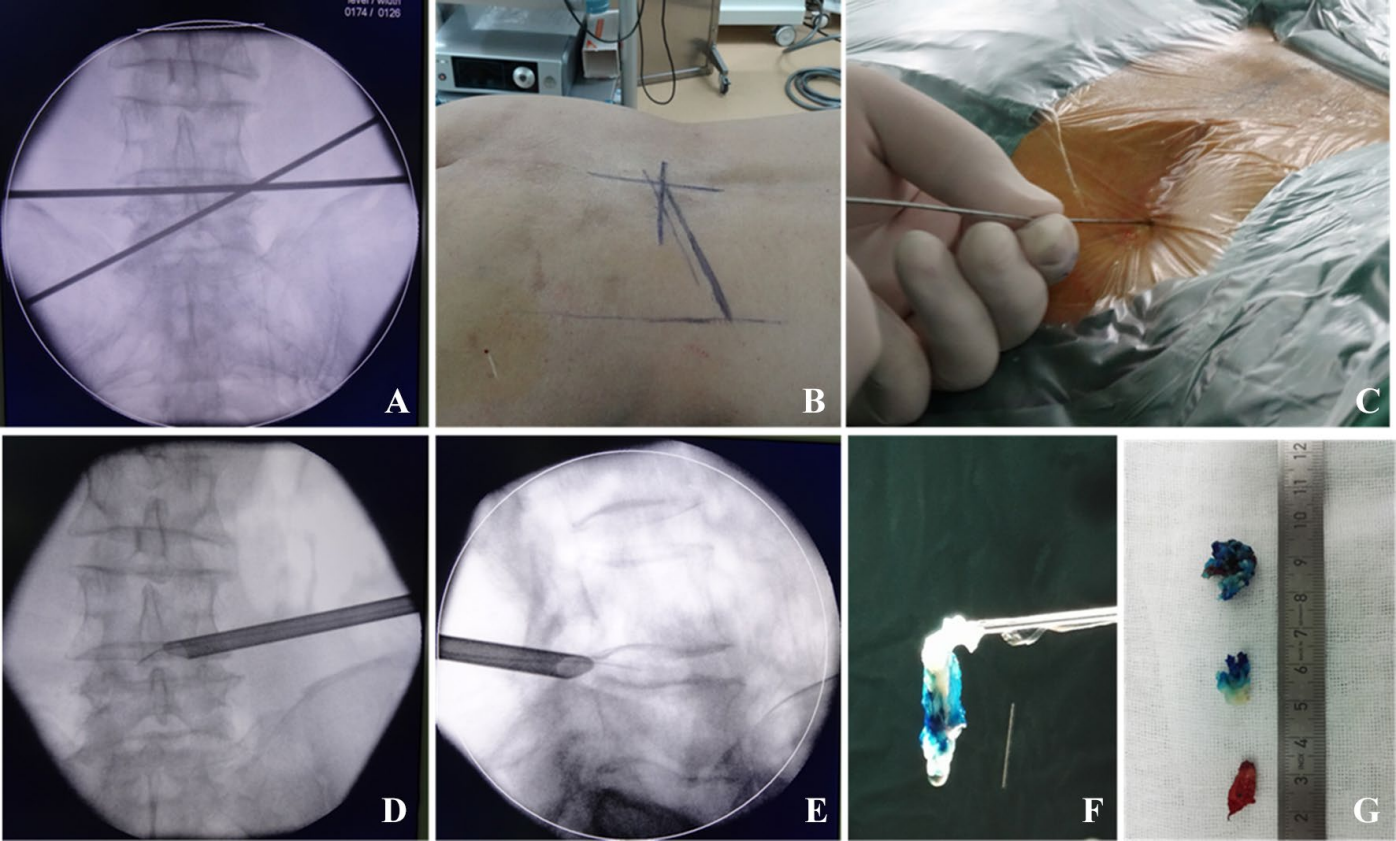
A representative case. **(A)** Puncture positioning; **(B)** skin marking; **(C)** local infiltration anaesthesia; **(D)** posterior perspective of the puncture position; **(E)** lateral perspective of the placement position; **(F)** lumbar 4/5 disc removal; **(G)** bone mass and intervertebral disc mass removal.

After successfully inducing anaesthesia, the patients were placed in the prone position. At 8 to 10 cm away from the spinal puncture, a puncture angle of 5–10° for L2/3 and L3/4 and a puncture angle of 15–30° for L4/5 and L5/S1 were chosen (Fig. 1A,B). Conventional disinfection cloths were applied with 0.5 lidocaine for infiltration anaesthesia (Fig. 1C). Then, the puncture needle entered the intervertebral disc directly through the Kambin triangle of the intervertebral foramen under fluoroscopy guidance (YESS approach), or a 1.5 mm Kirschner wire was positioned on the ventral side of the superior articular process and slid into the vertebral canal via the Kambin triangle through the intervertebral foramen (TESSYS approach). Staining and angiography of the intervertebral disc were performed with methylene blue and iodohydrin. After the positive stain and lateral fluoroscopy was confirmed to be correct, the skin was cut with the guide needle along the centre for 8 mm, and the working sleeve was placed after gradual expansion (the casing diameter was 7.8 mm, and the working sleeve could only be inserted after expanding and forming a lower intervertebral hole with a trephine). Staining and lateral fluoroscopy were performed to determine the position of the intervertebral space and puncture (Fig. 1D,E), and the yellow ligament and other tissues around the intervertebral foramen were gradually cleared to determine the position of the nerve roots, dural sac or intervertebral disc. According to these tissue structures, further microscopic localization was performed to ensure surgical safety. After the location of the protruded nucleus pulposus was clearly identified, degenerative nucleus pulposus tissue (Fig. 1F,G) that pressed on the nerves was removed, the spinal canal and nerve root alignment areas were carefully explored, residual nucleus pulposus tissue in the disc and spinal canal were cleared, and the dural sac and nerve root decompression were thoroughly observed. Finally, we use radiofrequency ablation electrodes to treat the annulus fibrosus and completely stop the bleeding; the working channel was removed, and the incision was sutured. A representative case is shown in Fig. 1.

### Statistical analysis

The statistical analyses in this study were performed with the statistical package SPSS, version 23.00 (SPSS Inc, Chicago, Illinois). Data are shown as the mean ± standard deviation (SD). Then, independent t-tests, Mann–Whitney U tests, chi-square tests, and Fisher’s exact tests were used to identify differences in clinical and radiological outcomes. P values < 0.05 were accepted as statistically significant.

### Ethics approval and consent to participate

This research was reviewed and approved by the medical ethics committees of our hospitals. Written informed consent was obtained from all participating patients.

## Results

**Figure 2:**
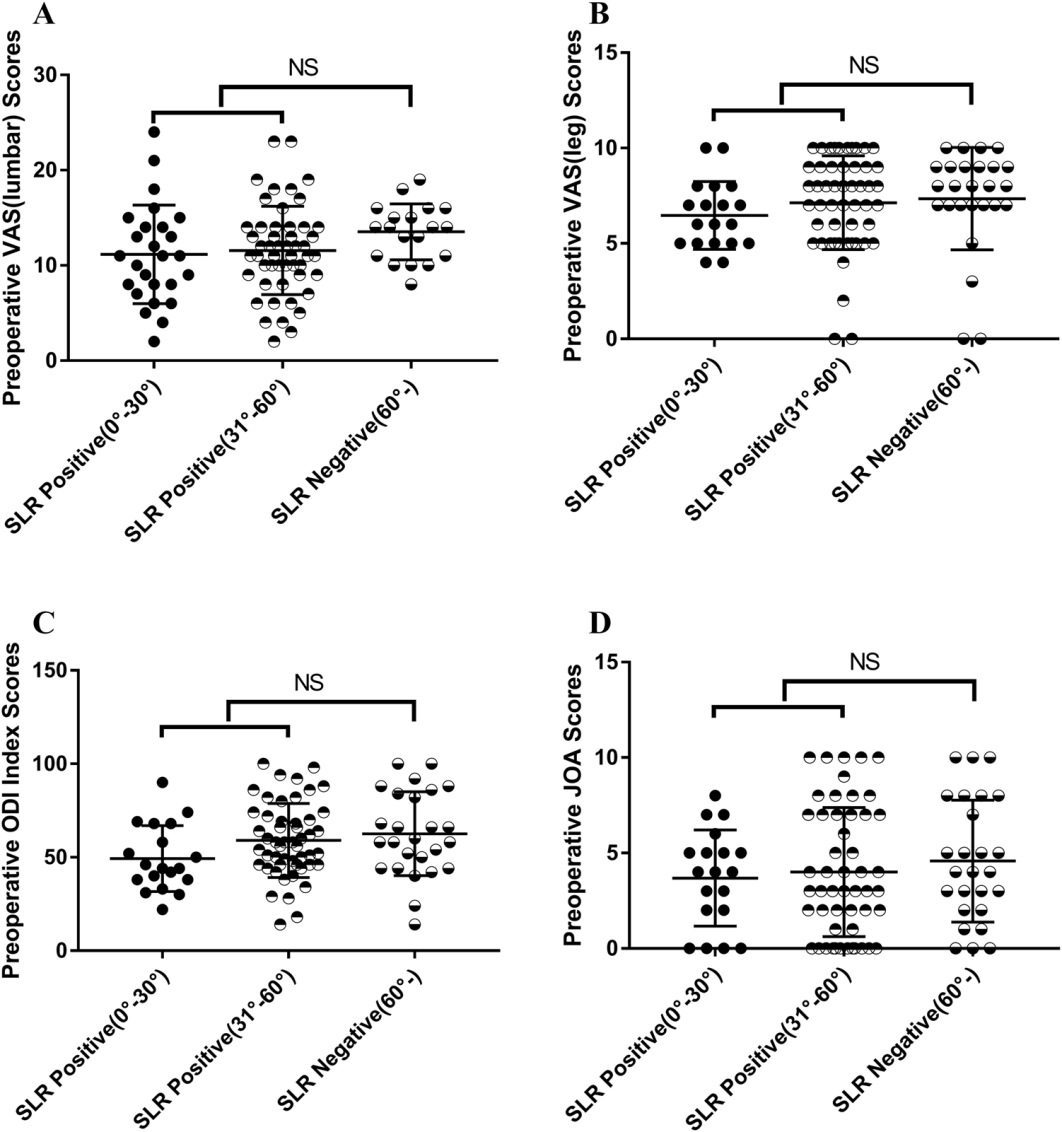
**(A–D)** The relationship between SLR and the VAS (lumbar/leg), ODI and JOA values.

This study involved 96 patients who underwent FETD surgery between August 2015 and September 2016, including 61 (63.5%) males and 35 (36.5%) females. With respect to the LDH sections, 2 (10.5%) were presented at L3–L4, 5 (26.3%) at L4–L5 and 12 (63.2%) at L5–S1 among SLR-positive (0°–30°) patients; 1 (2.0%) was presented at L3–L4, 25 (49.0%) at L4–L5 and 25 (49.0%) at L5–S1 among SLR-positive (0°–30°) patients; and 2 (7.7%) was presented at L3-L4, 13 (50.0%) at L4–L5 and 11 (42.3%) at L5–S1 among SLR-negative patients (Table 1). Of the 96 patients, 72 patients were SLR positive, and 24 patients were SLR negative. The BMI was 21.78 ± 6.30 for SLR-positive patients (0°–30°), 24.06 ± 3.27 for SLR-positive patients (31°–60°) and 23.61 ± 3.26 for SLR-negative patients. There was no statistically significant association between BMI and SLR (Table 1). All the surgeries were successful, and none of the patients underwent open surgery. In addition, there were no statistically significant differences among the three subpopulations [SLR positive (0°–30°), SLR positive (31°–60°) and SLR negative (61°–)], which is crucial because the three groups had similar starting characteristics (Table 2 and Fig. 2A–D).

**Table 1:** Demographic and baseline characteristics.

	Count n (%)	BMI (mean ± SD)	L3–L4 (%)	L4–L5 (%)	L5–S1 (%)
**SLR**					
Positive (0°–30°)	19 (21%)	21.78 ± 6.30	2 (10.5%)	5 (26.3 %)	12 (63.2%)
Positive (31°–60°)	51 (54%)	24.06 ± 3.27	1 (2.0%)	25 (49.0%)	25 (49.0%)
Negative (61°–)	26 (25%)	23.61 ± 3.26	2 (7.7%)	13 (50.0%)	11 (42.3%)
		*X*^2^, *p *= 0.464	*X*^2^, *p *= 0.492		

*SLR* straight leg raising test.

*Statistically significant (P < 0.05).

**Table 2:** Preoperative estimation of mean values of VAS, ODI and JOA for patients between SLR positive and negative (mean** ± **SD).

SLR	Preoperative VAS (lumbar)	Preoperative VAS (leg)	Preoperative ODI	Preoperative JOA
Total	4.09 ± 3.16	7.06 ± 2.40	58.08 ± 20.47	11.84 ± 4.56
Positive (0°–30°)	3.69 ± 2.52	6.47 ± 1.78	49.34 ± 17.62	13.53 ± 2.93
Positive (31°–60°)	4.00 ± 3.38	7.14 ± 2.46	59.02 ± 19.77	11.57 ± 4.64
Negative (61°–)	4.58 ± 3.19	7.35 ± 2.68	62.60 ± 22.48	11.15 ± 5.17
	*X*^2^, *p* = 0.614	*X*^2^, *p* = 0.114	*X*^2^, *p* = 0.065	*X*^2^, *p* = 0.076

*SLR* straight leg raising test, *VAS* visual analog scale, *ODI* Oswestry Disability Index, *JOA* Japanese Orthopedic Association.

*Statistically significant (P < 0.05).

**Figure 3:**
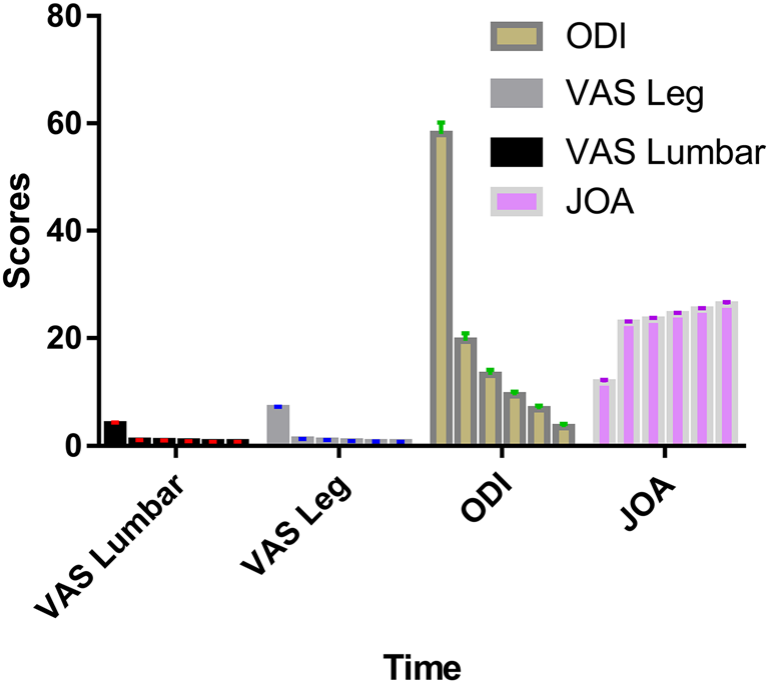
The VAS (lumbar/leg), ODI and JOA values in all patients preoperatively and at 1 day, 3, 6, 12, and 36 months postoperatively. *VAS* visual analogue scale, *ODI* Oswestry Disability Index, *JOA* Japanese Orthopaedic Association.

Ninety-six of the 118 patients were included in the follow-up. The remaining 22 patients were excluded at the following follow-up points: 2 at 1 month, 6 at 6 months, 5 at 12 months, and 9 at 36 months. Therefore, the 3-year follow-up rate was 83.36%. To evaluate postoperative efficacy, the internationally recognized ODI, JOA score, and VAS score were used to evaluate patients who were followed up for 3 years. All patients showed significant improvement after surgery. The ODI and VAS score (lumbar/leg) decreased in all patients and groups, and there was a statistically significant difference at each postoperative follow-up compared with the values before surgery (Fig. 3, * P < 0.05). Moreover, the increase in JOA after the operation was statistically significant compared with that before the operation (Fig. 3, * P < 0.05).

**Figure 4:**
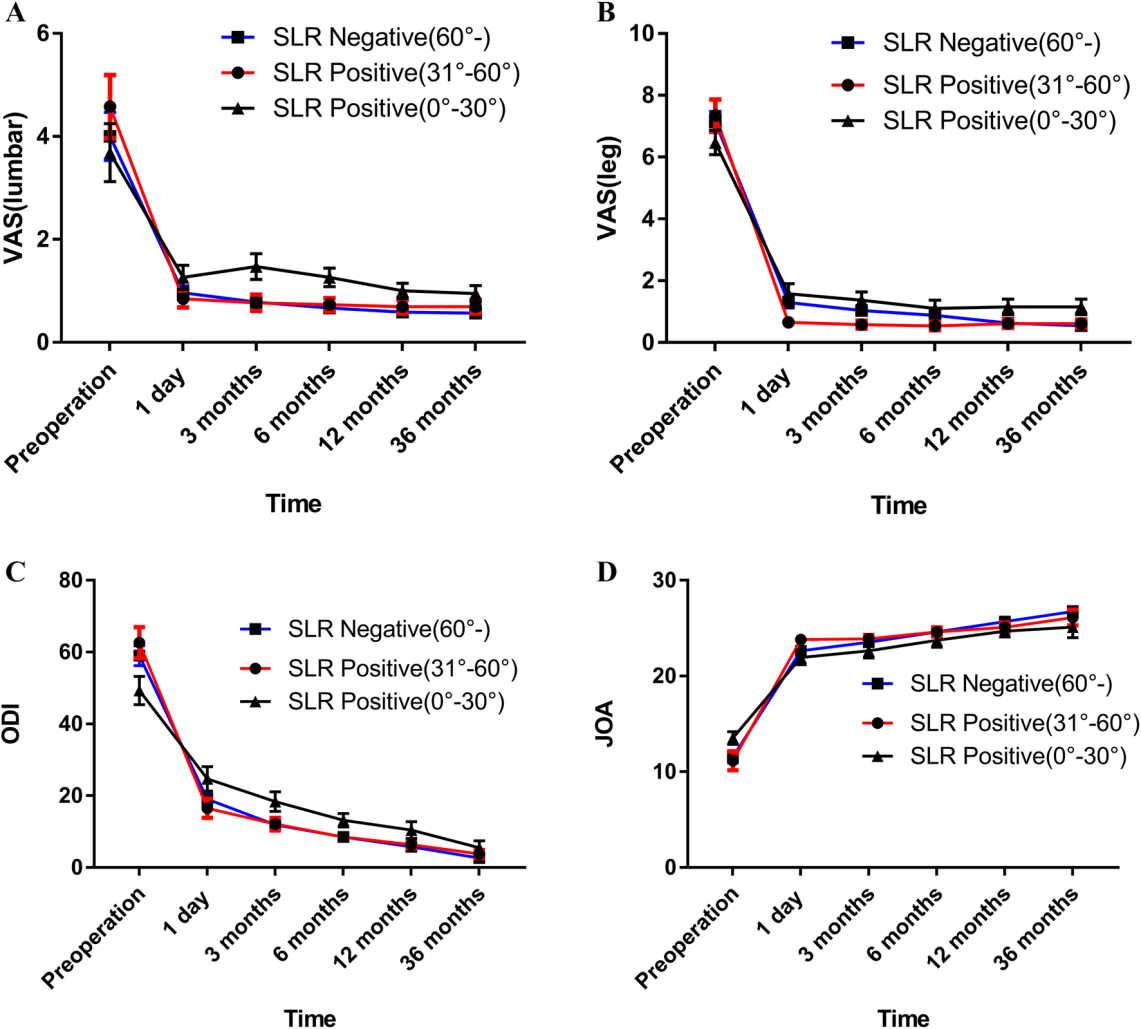
**(A)** The AS (lumbar) scores at 1 day, 3, 6, 12, 36 months postoperatively were related to SLR; **(B)** The VAS (leg) scores at 1 day, 3, 6, 12, 36 months postoperatively were related to SLR; **(C)** The ODI scores at 1 day, 3, 6, 12, 36 months postoperatively were related to SLR; **(D)** The JOA scores at 1 day, 3, 6, 12, 36 months postoperatively were related to SLR; *VAS* visual analogue scale, *ODI* Oswestry Disability Index, *JOA* Japanese Orthopaedic Association.

In terms of specific characterization, the VAS scores and ODIs at 1 day, 3, 6, 12 and 36 months after surgery were significantly lower than those before surgery, with statistically significant differences in both SLR-positive and SLR-negative patients. Moreover, the changes in VAS (leg), ODI and JOA values among SLR-negative patients were significantly higher at each follow-up point than those among SLR-positive patients, and the differences were statistically significant (Fig. 4A–D; Table 3). The changes in VAS (leg), ODI and JOA values at 1 day, 3, 6, 12 and 36 months postoperation compared with those before surgery showed statistically significant differences among SLR-positive (0°–30°), SLR-positive (31°–60°) and SLR-negative (61°–) patients. However, the changes in VAS (lumbar) score at 1 day, 3, 6, 12 and 36 months postoperation compared with those before surgery showed no statistically significant differences among SLR-positive (0°–30°), SLR-positive (31°–60°) and SLR-negative (61°–) patients (Fig. 4A–D; Table 3).

**Table 3:** Comparison of change of mean values for all chronological phases (2 phases each time) for patients between SLR positive and negative (mean** ± **SD).

SLR	Pre-op to 1d	Pre-op to 3 mo	Pre-op to 6 mo	Pre-op to 12 mo	Pre-op to 36 mo
**VAS (lumbar)**					
Positive (0°–30°)	2.42 ± 2.27	2.21 ± 2.70	2.42 ± 2.63	2.68 ± 2.60	2.78 ± 2.71
Positive (31°–60°)	3.04 ± 3.12	3.22 ± 3.13	3.33 ± 3.14	3.41 ± 3.16	3.43 ± 3.13
Negative (61 °–)	3.73 ± 3.21	3.81 ± 3.25	3.85 ± 3.17	3.88 ± 3.03	3.88 ± 3.03
	0.403	0.372	0.385	0.498	0.520
**VAS (leg)**					
Positive ( 0°–30° )	4.89 ± 1.73	5.11 ± 1.29	5.37 ± 1.46	5.32 ± 1.53	5.39 ± 1.54
Positive ( 31°–60° )	5.84 ± 2.49	6.10 ± 2.47	6.25 ± 2.46	6.51 ± 2.39	6.59 ± 2.38
Negative (61 °– )	6.69±2.60	6.77 ± 2.57	6.80 ± 2.59	6.73 ± 2.44	6.73 ± 2.44
	0.012*	0.006*	0.020*	0.010*	0.008*
**ODI**					
Positive ( 0°–30° )	24.77 ± 13.76	30.96 ± 14.97	36.19 ± 13.55	39.02 ± 16.55	44.92 ± 18.23
Positive ( 31°–60° )	39.99 ± 21.19	47.09 ± 19.45	50.55 ± 19.66	53.28 ± 19.19	56.41 ± 20.49
Negative (61 °– )	46.13 ± 20.37	50.55 ± 21.41	54.08 ± 20.79	56.20 ± 21.91	58.83 ± 22.32
	0.002*	0.001*	0.002*	0.008*	0.026*
**JOA**					
Positive ( 0°–30° )	8.42 ± 3.25	9.11 ± 3.11	10.21 ± 2.66	11.15 ± 2.32	11.61 ± 5.11
Positive ( 31°–60° )	11.08 ± 4.94	11.98 ± 4.71	13.04 ± 4.71	14.12 ± 4.60	15.18 ± 6.12
Negative (61 °– )	12.65 ± 4.72	12.73 ± 5.20	13.46 ± 4.93	13.92 ± 5.03	14.96 ± 6.48
	0.006*	0.012*	0.010*	0.010*	0.019*

*VAS* visual analog scale, *ODI* Oswestry Disability Index, *JOA* Japanese Orthopedic Association, *SLR* straight leg raising test.

*Statistically significant (P < 0.05).

## Discussion

FETD is considered a safe and effective method for the treatment of soft disc herniation. The advantages of this technique include preservation of the posterior disc structure, leading to less impact on the stability of the spine, and the effectiveness of this approach is similar to that of traditional open discectomy^10,11^. In recent years, FETD has undergone significant technological evolution, and the indications for FETD are also expanding^12–14^. Several randomized controlled studies have demonstrated that this new method showed great effectiveness in the treatment of LDH, which is consistent with our study^10,15,16^. While FETD brings great benefits to most patients, a small number of patients have poor outcomes or complications^17,18^. Therefore, further research is needed to explore the best indications for FETD to bring the greatest benefit to patients with LDH.

The SLR test serves as a valuable common examination method that can reflect the severity of lumbar disc herniation and the degree of nerve root compression to some extent^7^. Jonsson’s research showed that the SLR test has a strong correlation with various parameters that reflect the degree of pain. A positive postoperative SLR test was associated with an inferior outcome^8^. However, there are few studies on whether the SLR test could influence the operation, and there are few reports on which type of patients will benefit more from this kind of operation. Therefore, we designed this study to compare the prognostic differences between SLR-positive and SLR-negative patients who had LDH after FETD surgery. Our study showed that patients with FETD had a very good prognosis after surgery, and patients with very severe cases could independently care for themselves and gradually return to a normal life after surgery. This is similar to most reports on the efficacy of the FETD procedure^19,20^. There was no statistically significant difference in preoperative VAS (lumbar/leg) score, JOA score, or ODI among the three groups we selected, ensuring that the comparison was reliable and valuable. It is assumed that FETD is of great significance for the postoperative recovery of patients. The changes in the VAS (lumbar/leg), JOA and ODI values were greater in patients who were SLR negative than in patients who were SLR positive. This may show that SLR-negative patients benefitted more from the operation, although there was a statistically significant difference among the three groups in the main scores. In brief, FETD, as a novel technique, showed an excellent effect in the treatment of LDH. A study performed by Hyeun showed that FETD works well for all types of lumbar disc herniation, including extremely difficult cases^20^. Overall, there was a statistically significant difference in postoperative changes among SLR-positive (0°–30°), SLR-positive (31°–60°) and SLR-negative (61°–) patients. Therefore, there is an obvious difference in the effect of FETD surgery between SLR-positive and SLR-negative patients. Patients with a negative SLR might have less compression and less nerve damage, so the postoperative recovery is better. Patients with a negative SLR have better overall outcomes than patients with a positive SLR. FETD allows for enlargement of the neural foramen, and foraminal discectomy can achieve sufficient decompression, but when the nerve damage is severe, it may be difficult for the patient to recover completely. A positive SLR indicates extra tension on the nerve, increasing root ischaemia^21^. These may be the reasons for our result. However, it is still unclear what exactly caused SLR-negative patients to have better overall outcomes. This is our next study direction. The current study lacked a control group and with such a limited sample size, it was difficult to obtain valid prognostic considerations. In our future study, we will cooperate with other hospitals to increase the sample size and cooperate with other departments to include patients receiving non-surgical treatment.

Surgery-induced instability is a common consequence of OLM, which may occur at a rate as high as 22% after OLM^22^. Instability of the spine results from the removal of small lumbar muscles attached to the lamina and the removal of the facet joints. Patients undergoing FETD have less possibility of instability because these structures are preserved in this kind of surgery. A short-term retrospective study performed by Lee demonstrated that no patients in the PELD group developed instability, but 3.4% of patients who underwent OLM developed instability at the final follow-up^23^.

FETD has incomparable advantages over other technologies, including resection of only part of the superior articular process bone and preservation of most structures to avoid damaging the biomechanical structure of the spine, local anaesthesia, small incision, minimal bleeding, a short operation time and early ambulation^24–26^. There are three systematic reviews that suggest that FETD seems to be a safe and effective LDH intervention with similar clinical efficacy as traditional open microdiscectomy^27–29^.

This study has several limitations. For design reasons, there is no suitable control group because the purpose of this study is not to emphasize the possible advantages of FETD over other surgeries but to show the improvement of patients after receiving this surgery and the relationship of the outcomes with SLR. In addition, for many reasons, some patients were lost follow-up and did not complete the study.

While there have been many studies of FETD for LDH, none have looked at differences in improvement and postoperative recovery in terms of SLR. According to our findings, FETD seems to be a safe and effective technique. Patients who are SLR negative may have good outcomes according to VAS (leg). ODI and JOA values. In summary, there were significant differences among the three groups. Overall, these factors have important clinical impacts. However, whether SLR plays a role in the outcome requires exploration in large, multicentre, randomized, controlled studies. This study laid the foundation for a multicentre randomized controlled study.

## Conclusions

FETD showed great effectiveness in treating patients with lumbar disc herniation. The main scores included the VAS score (leg), ODI and JOA score, which showed that there were statistically significant differences among the three subpopulations treated by FETD. Patients who are SLR negative may receive greater benefit from FETD.
